# Preoperative evaluation of prostate cancer by ^68^Ga-PMSA positron emission tomography/computed tomography: comparison with magnetic resonance imaging and with histopathological findings

**DOI:** 10.1590/0100-3984.2022.0122-en

**Published:** 2023

**Authors:** Camila Edith Stachera Stasiak, Athos Cardillo, Sergio Altino de Almeida, Rosana Souza Rodrigues, Paulo Henrique Rosado de Castro, Daniella Braz Parente

**Affiliations:** 1 Universidade Federal do Rio de Janeiro (UFRJ), Rio de Janeiro, RJ, Brazil; 2 Instituto D’Or de Pesquisa e Ensino (IDOR), Rio de Janeiro, RJ, Brazil; 3 Universidade Federal Fluminense (UFF), Niterói, RJ, Brazil; 4 Universidade Federal do Estado do Rio de Janeiro (Unirio), Rio de Janeiro, RJ, Brazil

**Keywords:** Positron emission tomography/computed tomography, Multiparametric magnetic resonance imaging, Prostatic neoplasms, Tomografia por emissão de pósitrons/tomografia computadorizada, Ressonância magnética multiparamétrica, Neoplasias da próstata

## Abstract

**Objective:**

To evaluate the accuracy of preoperative positron emission tomography/computed tomography with ^68^Ga-labeled prostate-specific membrane antigen (^68^Ga-PSMA PET/CT) for staging prostate cancer and compare it with magnetic resonance imaging (MRI) using histopathology of surgical specimens as the gold standard.

**Materials and Methods:**

In this retrospective study, 65 patients with prostate cancer were analyzed.

**Results:**

The accuracy of ^68^Ga-PSMA PET/CT for tumor detection was 95%, and that of MRI was 91%. There was no difference between ^68^Ga-PSMA PET/CT and MRI regarding localization of the lesion. The sensitivity of ^68^Ga-PSMA PET/CT for detecting extraprostatic extension was quite low (14%). For detection of seminal vesicle invasion, ^68^Ga-PSMA PET/CT showed a sensitivity of 57% and accuracy of 91%. There was a moderate correlation between the maximum standardized uptake value (SUVmax) and the serum level of prostate-specific antigen (*p* < 0.01; ρ = 0.368) and between the SUVmax and the International Society of Urological Pathology (ISUP) grade (*p* < 0.01; ρ = 0.513).

**Conclusion:**

^68^Ga-PSMA PET/CT is a promising tool for detecting and evaluating the primary tumor, which can alter the staging and management of the disease.

## Abstract (pt)

**Objetivo:**

Avaliar a acurácia da tomografia por emissão de pósitrons/tomografia computadorizada com PSMA (PET-PMSA) pré-operatória para estadiamento do câncer de próstata e compará-la com a ressonância magnética (RM) utilizando o histopatológico cirúrgico como padrão ouro.

**Materiais e Métodos:**

Neste estudo retrospectivo foram analisados 65 pacientes com câncer de próstata.

**Resultados:**

A acurácia da PET-PSMA para a detecção tumoral foi de 95% e a da RM foi de 91%. Não houve diferença entre a PET-PSMA e a RM quanto à localização da lesão. A PET-PSMA apresentou baixa sensibilidade (14%) para detecção de extensão extraprostática em comparação ao histopatológico. Para detecção de invasão de vesícula seminal, a PET-PSMA apresentou sensibilidade de 57% e acurácia de 91% em comparação ao histopatológico. Houve correlação moderada entre o SUVmax e o PSA (*p* < 0,01; ρ = 0,368) e entre o SUVmax e o ISUP (*p* < 0,01; ρ = 0,513).

**Conclusão:**

A PET-PSMA é uma ferramenta promissora para detecção e avaliação do tumor primário, alterando o estadiamento e a conduta do paciente.

## INTRODUCTION

The estimated incidence of new cases of prostate cancer in Brazil for the 2020-2022 (three-year) period is 66,000, which corresponds to an estimated risk of 63 new cases per 100,000 men^(1)^. Prostate cancer has a broad spectrum of behavior, ranging from slow-growing indolent tumors to rapidly progressive, aggressive disease. Given the high prevalence of the disease, early and effective diagnosis and staging are key factors in choosing the most appropriate therapeutic strategy and in the prognosis of the affected patients.

Multiparametric magnetic resonance imaging (mpMRI) of the prostate is currently the best imaging method for the local staging of prostate cancer, allowing tumor localization, detection of extraprostatic disease, and evaluation of invasion of the seminal vesicles or adjacent organs, as well as detection of bone metastases in the pelvis^(2,3)^. However, MRI is less sensitive for the diagnosis of lymph node metastases^(4,5)^.

Hybrid imaging of positron emission tomography/computed tomography with ^68^Ga-labeled prostate-specific membrane antigen (^68^Ga-PSMA PET/CT) is an increasingly used tool in the detection of biochemical recurrence. For the staging of prostate carcinoma, recent studies have shown that ^68^Ga-PSMA PET/CT has considerable potential, as does as PSMA-PET/CT with other radiotracers, especially in patients with intermediateor high-risk disease, as well as in those with negative findings, inconclusive findings, or oligometastatic disease on conventional imaging examinations^(6)^. A ^68^Ga-PSMA PET/CT scan can accurately localize the index tumor in the prostate^(7,8)^. In the detection of affected lymph nodes, some studies have suggested that ^68^Ga-PSMA PET/CT is superior to MRI for detecting lymph node metastases^(9-12)^. Compared with tomography of the abdomen and pelvis and bone scintigraphy, examinations traditionally performed in staging, ^68^Ga-PSMA PET/CT presents greater sensitivity for distant metastases and a higher rate of change in staging^(13)^. However, few studies have evaluated the use of ^68^Ga-PSMA PET/CT in the detection and evaluation of early-stage prostate cancer.

In this study, we evaluated patients with biopsy-confirmed prostate cancer who had undergone preoperative staging that included ^68^Ga-PSMA PET/CT and mpMRI. The objectives were to evaluate the accuracy of ^68^Ga-PSMA PET/CT for the detection and staging of prostate cancer, using histopathological findings as the gold standard; to evaluate the correlation between PSMA uptake and serum levels of prostate-specific antigen (PSA) and with histopathological criteria of aggressiveness of the surgical specimen; and to compare ^68^Ga-PSMA PET/CT and mpMRI in terms of their accuracy for the detection and localization of prostate cancer, as well as for the detection of extraprostatic extension and seminal vesicle invasion.

## MATERIALS AND METHODS

This was a retrospective, observational, cross-sectional study. The study was approved by the local research ethics committee and was conducted in accordance with national and international resolutions, as established in Brazilian National Health Council Resolution no. 466 (December 12, 2012) and in complementary statements issued by the Council and by the Brazilian National Health Ministry, as well as in the Declaration of Helsinki, all revisions and amendments thereto, and in the Document of the Americas. Because of the retrospective nature of the study, the requirement for informed consent was waived.

We included 65 patients who were followed between 2017 and 2020. The inclusion criteria were having biopsy-confirmed prostate cancer with an indication for prostatectomy and having undergone preoperative ^68^Ga-PSMA PET/CT and mpMRI of the prostate. Patients with a history of another malignancy were excluded, as were those who had undergone other treatments for prostate cancer prior to surgery. All of the patients evaluated underwent prostatectomy, with or without lymphadenectomy, according to the indication and practice of the attending physician.

All mpMRI examinations were performed in 1.5-T or 3.0-T scanners, including T2-weighted, diffusion-weighted, and dynamic contrast-enhanced imaging. All findings are reported in accordance with the guidelines established in the Prostate Imaging Reporting and Data System (PI-RADS), version 2.1^(14,15)^. The examinations were analyzed by a radiologist, with 18 years of experience, who was blinded to clinical and histopathological data.

The PET/CT images were acquired 60 min after intravenous injection of 1.8-2.2 MBq of ^68^Ga-PSMA-11, as previously recommended^(16)^. Excluding those in whom it was contraindicated, all of the patients received 20 mg of intravenous furosemide 20 min after administration of the radiopharmaceutical, together with intravenous hydration. All images were acquired from the skull vertex to the mid-thigh, without administration of iodinated contrast. The ^68^Ga-PSMA PET/CT scans were analyzed by a nuclear physician, with three years of experience, who was blinded to the clinical and histopathological data. All findings are reported in accordance with the joint guidelines established by the European Association of Nuclear Medicine and the (American) Society of Nuclear Medicine and Molecular Imaging^(16)^.

Categorical variables are expressed as absolute and relative frequencies, whereas continuous variables are expressed as mean and standard deviation if they were normally distributed or as medians and interquartile ranges if they were not, as determined by using the Shapiro-Wilk test. Accuracy was calculated, and its significance was determined through the use of tests of proportion. Variables were analyzed with the nonparametric paired Wilcoxon test, Spearman’s correlation test, or McNemar’s test. McNemar’s test was applied to quantify agreement between dependent categorical variables as an alternative to the chi-square test, which presupposes that the variables are independent. In McNemar’s test, *p* > 0.05 indicates that the variables agree. The level of statistical significance was set at 5%. Statistical analysis was performed with the program R, version 3.6.1 (The R Foundation for Statistical Computing, Vienna, Austria).

## RESULTS

Table 1 shows clinical, pathological, PI-RADS, and SUVmax data related to the patients evaluated.

**Table 1:** Clinical and pathological data related to the patients evaluated.

Variable			(N = 65)
Age (years), mean ± SD			69.3 ± 6.2
Serum PSA (ng/mL), median (IQR)			6.8 (4.5-12)
Prostate size in the surgical specimen (g), median (IQR)			49 (33-68)
Tumoral volume in the surgical specimen (%), median (IQR)			25 (15-35)
Scores and corresponding grades, n (%)	Preoperative biopsy	Surgical specimen	
Gleason 3+3 - ISUP 1	8 (12.5)	2 (3.1)	
Gleason 3+4 - ISUP 2	32 (50)	36 (55.4)	
Gleason 4+3 - ISUP 3	14 (21.9)	15 (23.1)	
Gleason 4+4 - ISUP 4	6 (9.4)	2 (3.1)	
Gleason 4+5 - ISUP 5	2 (3.1)	8 (12.3)	
Gleason 5+4 - ISUP 5	0	2 (3.1)	
Gleason 5+5 - ISUP 5	2 (3.1)	0	
Underwent lymphadenectomy, n (%)			31 (47.7)
Negative lymph nodes			22 (71.0)
Positive lymph nodes			9 (29.0)
No lymphadenectomy, n (%)			34 (52.3)
Invasion of seminal vesicles, n (%)			15 (23.4)
Extraprostatic extension, n (%)			7 (10.9)
PI-RADS category on mpMRI, n (%)			
PI-RADS 2			6 (9.2)
PI-RADS 3			4 (6.2)
PI-RADS 4			29 (44.6)
PI-RADS 5			26 (40.0)
SUVmax on ^68^Ga-PSMA PET/CT, median (IQR)			7.8 (5.7-14.1)

SD, standard deviation; IQR, interquartile range.

### ^68^Ga-PSMA PET/CT

**Figure 1:**
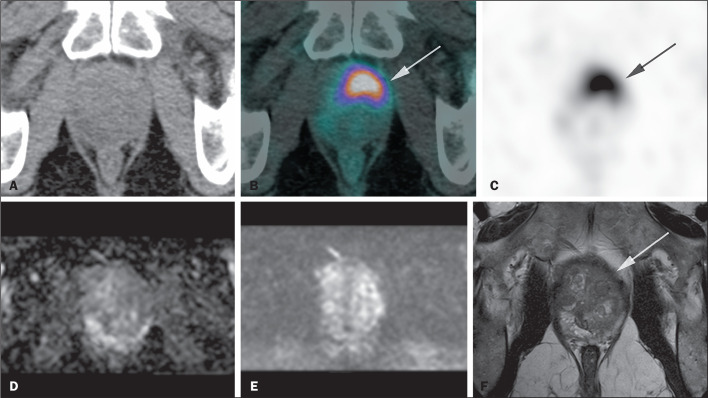
A 67-year-old patient with a serum PSA of 23.1 ng/mL. The image shows intense radiopharmaceutical uptake in an area centered on the anterior midline and to the left in the apical portion of the prostate, affecting the transition zone, with an SUVmax of 14.9 (A: CT; B: ^68^Ga-PSMA PET + CT fusion; C: ^68^Ga-PSMA PET/CT), which corresponds to the area at the apex to the left of the transition zone, with a discrete hypointense signal on T2-weighted sequences, without significant restricted diffusion on diffusion-weighted imaging and without alteration in the perfusion study on mpMRI (D: apparent diffusion coefficient map; E: diffusion-weighted image; F: T2-weighted image).

On ^68^Ga-PSMA PET/CT, 61 (94%) of the 65 patients showed high-intensity radiotracer uptake in the prostate: in the peripheral zone in 48 (74%), in the transition zone five (8%), and in both zones in eight (12%). Figure 1 shows a patient with a focus of intense radiotracer uptake in the transition zone. We identified extraprostatic extension in six patients (9%) and seminal vesicle invasion in eight (12%). Eight patients (12%) had one or more lymph nodes with high-intensity radiotracer uptake on ^68^Ga-PSMA PET/CT.

Bone uptake foci were detected on ^68^Ga-PSMA PET/CT in five patients (8%) . However, four of those patients had one or more foci of discrete radiotracer uptake, without corresponding morphological changes on the CT images, which were considered probable benign lesions . One patient had a focus, with an SUVmax of 3.5, in vertebral body T1 and another, with an SUVmax of 8.8, in the right sixth rib, both corresponding to small sclerotic lesions on CT. No metastases to the lungs, liver, or other viscera were identified in any of the patients.

The mean SUVmax at the suspected index lesion was 7.8 (5.7-14.1). The median International Society of Urological Pathology (ISUP) grade was 2 (mean, 2.7 ± 1.2). There was a statistically significant correlation between the SUVmax and the serum PSA level (*p* < 0.01) and between the SUVmax and the ISUP grade (*p* < 0.01). Spearman’s correlation coefficient was moderate in the correlation between the SUVmax and the serum PSA level (ρ = 0.368) and in that between the SUVmax and the ISUP grade (ρ = 0.513).

### mpMRI

In 59 (91%) of the 65 patients, mpMRI of the prostate identified at least one lesion classified as PI-RADS 3 or greater: in the peripheral zone in 43 (66%), in the transition zone in 11 (17%), and in both zones in five (8%). Extraprostatic extension was identified in 19 patients (29%), and seminal vesicle invasion was identified in 12 (18%). Suspicious lymph nodes were identified in six patients (9%). No bone lesions suspicious for metastasis were detected on any of the mpMRI scans.

### Histopathology

In the histopathology of the surgical specimen, tumor-free margins were observed in 49 patients (75%). In 58 patients (89%), there was perineural invasion. The ISUP grade was significantly higher in the surgical specimen than in the preoperative biopsy (*p* < 0.01). Of the 65 patients, 31 (48%) underwent lymphadenectomy, in which a total of 345 lymph nodes, an average of 11.1 lymph nodes per patient, were resected. Of those 345 lymph nodes, 24 (7%), in nine patients, were positive for lymph node metastasis.

### Comparison between imaging modalities

In 45 patients (69%), ^68^Ga-PSMA PET/CT and mpMRI provided similar results in terms of the laterality of the suspicious lesion, with no significant difference between the two modalities (*p* = 0.14).

Regarding the localization of the tumor in the peripheral or transitional zone, ^68^Ga-PSMA PET/CT showed a sensitivity of 72%, a specificity of 85%, and an accuracy of 74% when compared with mpMRI, and the difference between the two modalities was also not significant (*p* = 0.11). As for localization in the apex, middle third, or base of the prostate, ^68^Ga-PSMA PET/CT showed a sensitivity of 62%, a specificity of 94%, and an accuracy of 59% in comparison with mpMRI, a difference that was also not statistically different.

Other factors that could be taken into account are the presence of extraprostatic extension and seminal vesicle invasion. For the detection of extraprostatic extension, ^68^Ga-PSMA PET/CT showed a sensitivity of 32%, albeit with 100% specificity and an accuracy of 80%, when compared with mpMRI, whereas it showed a sensitivity of 67%, a specificity of 100%, and an accuracy of 94% for the detection of seminal vesicle invasion.

### Comparison between imaging and histopathological studies

For tumor detection (i.e., whether the examination was positive or negative), in comparison with histopathology, ^68^Ga-PSMA PET/CT showed a sensitivity of 95% and mpMRI showed a sensitivity of 91%. The examination was positive in 61 patients on ^68^Ga-PSMA PET/CT and in 59 patients on mpMRI.

For determining the laterality of the tumor, in relation to the preoperative biopsy, mpMRI had an overall accuracy of 56%, with a sensitivity of 59% and specificity of 83%. The biopsy identified bilateral disease in 24 patients, compared with 11 patients for mpMRI. For identifying bilateral disease, ^68^Ga-PSMA PET/CT had an overall accuracy of 51% compared with the biopsy, with a sensitivity of 53% and specificity of 79%, identifying such disease in 16 patients.

**Figure 2:**
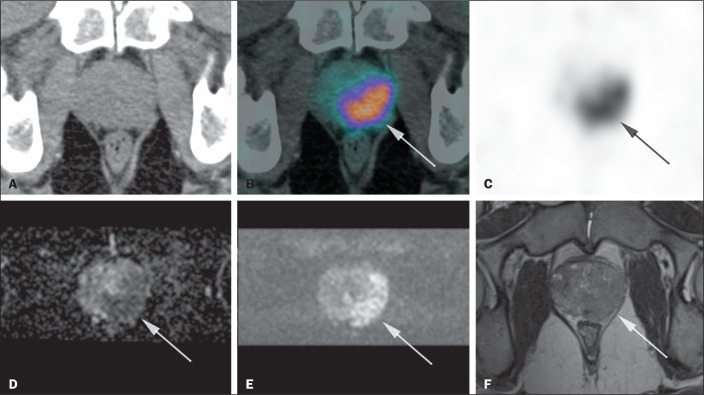
A 64-year-old patient with a serum PSA of 12 ng/mL, who presented with an altered digital rectal examination on the left. The image shows increased radiopharmaceutical uptake in a lesion predominantly in the left half of the prostate, from the apex to the base, with an SUVmax of 21.9 (A: CT; B: ^68^Ga-PSMA PET/CT + CT fusion; C: ^68^Ga-PSMA PET/CT), which corresponds to an infiltrative lesion affecting the peripheral and transition zones, from the apex to the base, measuring 4.3 cm on its longest axis, showing low signal intensity on T2-weighted imaging and restricted diffusion on diffusion-weighted imaging, with extraprostatic extension, categorized as PI-RADS 5 on mpMRI (D: apparent diffusion coefficient map; E: diffusion-weighted image; F: T2-weighted image).

For detecting extraprostatic extension, in comparison with histopathology, mpMRI and ^68^Ga-PSMA PET/CT had low sensitivity (29% and 14%, respectively). Figure 2 shows an example of a prostatic lesion with extraprostatic extension. For detecting seminal vesicle invasion, in comparison with histopathology, mpMRI showed an accuracy of 94% and ^68^Ga-PSMA PET/CT showed an accuracy of 91%. Those data are shown in Table 2.

**Table 2:** Comparison between imaging and histopathology in relation to extraprostatic extension and seminal vesicle invasion (N = 65).

Finding	Stage		
	T2 (n = 43)	T3a (n = 7)	T3b (n = 15)
Extraprostatic extension, n (%) mpMRI^*^			
No	38 (88.4)	5 (71.4)	3 (20.0)
Yes	5 (11.6)	2 (28.6)	12 (80.0)
^68^Ga-PSMA PET/CT^†^			
No	43 (100)	6 (85.7)	10 (66.7)
Yes	0	1 (14.3)	5 (33.3)
Seminal vesicle invasion, n (%)			
mpMRI^‡^ No	42 (97.7)	7 (100)	4 (26.7)
Yes	1 (2.3)	0	11 (73.3)
^68^Ga-PSMA PET/CT^§^			
No	43 (100)	7 (100)	7 (46.7)
Yes	0	0	8 (53.3)

T2, histopathologically confined to the prostate; T3a, extraprostatic extension; T3b, seminal vesicle invasion.

* Sensitivity, 28.6%; specificity, 70.2%; accuracy, 65.6%-*p* = 0.02 vs. histopathology.

† Sensitivity, 14.3%; specificity, 91.2%; accuracy, 82.8%-*p* = 0.99 vs. histopathology.

‡ Sensitivity, 78.6%; specificity, 98.0%; accuracy, 93.8%-*p* = 0.62 vs. histopathology.

§ Sensitivity, 57.1%; specificity, 100%; accuracy, 90.6%-*p* = 0.05 vs. histopathology.

Of the 65 patients, 31 (48%) underwent lymphadenectomy. Of those 31 patients, nine (29%) had lymph node metastasis on histopathology. Eight (12%) of the 65 patients had one or more lymph nodes with high-intensity radiotracer uptake on ^68^Ga-PSMA PET/CT, and six (9%) had suspicious lymph nodes on mpMRI. Of the nine patients who had lymph node metastasis on histopathology, four (44%) were correctly identified on ^68^Ga-PSMA PET/CT and five (56%) were not. Among the eight patients who had high-intensity lymph node uptake on ^68^Ga-PSMA PET/CT, the histopathology did not identify positive lymph nodes in four (50%). All patients with positive lymph nodes on ^68^Ga-PSMA PET/CT underwent lymphadenectomy. Of the six patients with suspicious lymph nodes on mpMRI, two (33%) did not have positive lymph nodes on histopathology, two (33%) were not submitted to lymphadenectomy, and two (33%) had positive lymph nodes correctly identified on mpMRI and histopathology.

## DISCUSSION

For diagnosing patients with clinically significant prostate cancer, ^68^Ga-PSMA PET/CT and mpMRI both proved to be highly sensitive. There was no statistically significant difference between ^68^Ga-PSMA PET/CT and mpMRI in relation to the localization of the lesion in the transition or peripheral zone; the localization of the lesion in the base, middle third, or apex of the prostate; or the laterality of the lesion. Therefore, both methods proved to be appropriate for the evaluation of patients with suspected prostate cancer.

For tumor detection, in comparison with histopathology, we found that ^68^Ga-PSMA PET/CT had a sensitivity of 95% and that mpMRI had a sensitivity of 91%. Studies comparing ^68^Ga-PSMA PET/CT with histopathology have produced conflicting results. As in the present study, Berger et al.^(17)^ found that the prostate tumor detection rate was 100% for ^68^Ga-PSMA PET/CT and 94% for mpMRI. In contrast, Perera et al.^(10)^ reported a prostate tumor detection rate of 40% for ^68^Ga-PSMA PET/CT. In the study carried out by Pallavi et al.^(18)^, the sensitivity of ^68^Ga-PSMA PET/CT for prostate tumor detection was 86.2%, compared with 68.6% for mpMRI, lower than the values obtained in our study. Despite the discrepancies in the literature, in clinical practice, a ^68^Ga-PSMA PET/CT or mpMRI examination with a positive result for clinically significant prostate cancer calls for investigation with a prostate biopsy.

For determining the laterality of the tumor in the prostate, in comparison with the preoperative biopsy, we found that ^68^Ga-PSMA PET/CT had an accuracy of 51% and mpMRI had an accuracy of 56%. A possible explanation for the low accuracy of these methods in our study is the comparison with the biopsy, which may not have included the clinically significant lesion. This hypothesis is supported by the increase in the histopathological grade detected in the surgical specimen when compared with the biopsy. In addition, due to the retrospective nature of the study, it was not possible to access the entire (whole-mount) surgical specimen, which limited the evaluation of the location of the index lesion on histopathology. A prostate tumor is an infiltrative neoplasm that affects the prostate diffusely, and most histopathological analyses of the surgical specimen reflect the bilaterality of this involvement, without highlighting the index lesion.

Regarding localization of the tumor in the peripheral zone or in the transition zone, we found that ^68^Ga-PSMA PET/CT had an accuracy of 74% when compared with mpMRI. Although mpMRI typically assesses zonal delimitation more precisely, we did not find a significant difference between ^68^Ga-PSMA PET/CT and mpMRI in our study. We also found no significant difference between the two modalities regarding the localization of the tumor in the base, middle third, or apex of the prostate. Kalapara et al.^(7)^ compared ^68^Ga-PSMA PET/CT and mpMRI with the histopathology of the surgical specimen, analyzing the laterality, the prostate third, and the zone for the localization of the index lesion. As in our study, those authors found no difference between the two modalities: ^68^Ga-PSMA PET/CT correctly located 91% of index tumors, and mpMRI correctly located 89%. Yilmaz et al.^(8)^ showed that ^68^Ga-PSMA PET/CT was able to localize the tumor in 70.8% of the patients and mpMRI was able to localize the tumor in 54.2%, proportions lower than those obtained in our study. These discrepancies in the literature could be due to factors such as lack of standardization in the criteria for the interpretation of PSMA-PET, differences in the level of reader experience, and differences among the populations evaluated. Despite the small variation in the results in the literature, there is a tendency for there to be no significant difference between PSMA-PET and MRI in the localization of the index lesion, which is in agreement with our data.

Our study evaluated prostate tumor detection, regardless of the index lesion and secondary lesions. In a similar manner, Berger et al.^(17)^ showed that ^68^Ga-PSMA PET/CT and mpMRI both had a high index lesion detection rate (100% and 94%, respectively), and that ^68^Ga-PSMA PET/CT detected a greater proportion of additional lesions in the prostate than did MRI (93.5% vs. 51.6%). For the localization of the lesion, the authors found that ^68^Ga-PSMA PET/CT showed greater sensitivity than did mpMRI (81.1% vs. 64.8%), with similar specificity. As in our study, Donato et al.^(19)^ showed that ^68^Ga-PSMA PET/CT and mpMRI had similar sensitivity in detecting the index prostate tumor (93% vs. 90%), although they found that ^68^Ga-PSMA PET/CT had greater sensitivity than did mpMRI for detecting bilateral lesions (42% vs. 21%) and multifocal lesions (34% vs. 19%). Therefore, the index lesion and additional lesions are well identified by ^68^Ga-PSMA PET/CT.

Other aspects evaluated in tumor staging are extraprostatic extension and seminal vesicle invasion. The literature presents conflicting data on these assessments. Our data show that ^68^Ga-PSMA PET/CT had low sensitivity for detecting extraprostatic extension when compared with histopathology and with MRI (14% and 32%, respectively), which is expected given the characteristics of the former modality. However, ^68^Ga-PSMA PET/CT had high specificity, showing that when it suggests extraprostatic extension, it is probably true. Similarly, Yilmaz et al. ^(8)^ showed that mpMRI has better accuracy than does 68Ga-PSMA PET/CT for detecting extraprostatic extension (87.5% vs. 66.7%). In contrast, Chen et al.^(20)^ found that mpMRI showed lower sensitivity for detecting extraprostatic extension than did ^68^Ga-PSMA PET/CT (54% vs. 78%). The lack of standardization in the criteria for the evaluation of extraprostatic extension by PSMA-PET could be a limiting factor in the comparison across studies.

For the detection of seminal vesicle invasion, we found the accuracy of ^68^Ga-PSMA PET/CT to be 91% compared with histopathology and 94% compared with MRI, suggesting that ^68^Ga-PSMA PET/CT has considerable potential for that evaluation. Different than in our study, Yilmaz et al.^(8)^ showed that the accuracy of mpMRI for the detection of seminal vesicle invasion was better than was that of ^68^Ga-PSMA PET/CT (95.8% vs. 87.5%). However, in agreement with our data, Chen et al.^(20)^ reported no significant difference between ^68^Ga-PSMA PET/CT and mpMRI in terms of the detection of seminal vesicle invasion. Therefore, further studies are needed in order to better assess the accuracy of imaging methods for the detection of that feature of prostate cancer.

Our study showed a moderate positive correlation between the degree of PSMA uptake, as assessed by the SUVmax, and tumor aggressiveness, as assessed by the ISUP grade. We also identified a moderate positive correlation between the SUVmax and serum PSA. Ergül et al.^(21)^, in agreement with our data, also reported that a higher Gleason score and higher serum PSA translate to a higher SUVmax and greater tumor aggressiveness.

The detection of lymph node metastases influences the treatment and prognosis of prostate cancer. In the literature, the sensitivity and specificity of ^68^Ga-PSMA PET/CT and mpMRI for detecting such metastases vary across studies, although some have suggested that ^68^Ga-PSMA PET/CT is superior^(10-12)^. Other studies of ^68^Ga-PSMA PET/CT have shown that its sensitivity and specificity vary widely, ranging from 38.2% to 87.0% and from 90.9% to 100%, respectively^(12,13,22-27)^. In the present study, we did not evaluate the sensitivity and specificity of ^68^Ga-PSMA PET/CT and mpMRI for the detection of lymph node metastases, because of the small number of patients in whom there were positive lymph nodes on imaging and the small number who underwent lymphadenectomy, as well as the lack of information regarding the localization of the lymph nodes that were positive on histopathology. Therefore, further studies are needed to better clarify the accuracy of ^68^Ga-PSMA PET/CT and mpMRI in the evaluation of metastatic lymph nodes.

In our study, there were four cases in which ^68^Ga-PSMA PET/CT and the histopathology both showed positive lymph nodes and two cases in which MRI and the histopathology both showed positive lymph nodes. The mean spatial resolution of ^68^Ga-PSMA PET/CT is 4-5 mm^(28)^, which could explain the fact that some patients had lymph nodes that did not show high-intensity uptake on ^68^Ga-PSMA PET/CT but were positive on histopathology. However, the lymph nodes with high-intensity uptake that were negative on histopathology might represent benign (inflammatory or reactive) processes in lymph nodes or lymph nodes in chains that are not typically resected, such as the mesorectal chain. Likewise, lymph nodes that are suspicious on MRI and are not confirmed as metastatic on histopathology might represent benign or unresected lymph nodes. Lymph nodes that are negative on ^68^Ga-PSMA PET/CT and MRI but positive on histopathology are generally small, with a mean diameter of 4.0-5.5 mm^(27,29)^. Franklin et al.^(30)^ reported that, in 32.8% of patients, lymph nodes that were negative on ^68^Ga-PSMA PET/CT were positive on histopathology. Those patients had tumors that were more aggressive (ISUP grade 4 or 5) or were categorized as PI-RADS 5 on mpMRI. Therefore, in the case of an aggressive tumor, even with a negative result for lymph node metastasis on ^68^Ga-PSMA PET/CT and mpMRI, extended lymphadenectomy may be beneficial.

In the current study, foci of intense bone radiotracer uptake were found in five patients. However, those findings did not contraindicate surgical treatment. Postoperative management data, such as the initiation of hormone blocking therapy or radiotherapy for bone lesions, were not analyzed. To date, there are no data contraindicating curative treatment in patients with oligometastatic disease found on PET/CT-PSMA^(31)^. Similarly, in the study conducted by Hofman et al.^(13)^, 2.7% of the patients had metastatic disease and underwent radical treatment.

In addition to detecting bone disease, ^68^Ga-PSMA PET/CT can detect foci of metastasis to viscera such as the lungs and liver^(32)^. None of the patients in our sample had visceral metastasis. However, that might be due to the retrospective nature of the study, in which we analyzed only patients who underwent radical prostatectomy.

Our study has some limitations due to its retrospective nature. The biggest limitation was the lack of comparison with the entire (whole-mount) surgical specimen, which can hinder the localization of the index lesion, as well as reducing the accuracy of the ^68^Ga-PSMA PET/CT and mpMRI. However, the detection of a tumor, which was evaluated in our study, has a greater impact on clinical practice than does its exact localization in the prostate. In addition, we were not able to discriminate the lymph node chain in the histopathology for reliable comparison with the imaging findings, which limited the evaluation of lymph node metastasis, and there were few patients with positive lymph nodes, as well as few patients who underwent lymphadenectomy. Furthermore, we were not able to assess the level of interobserver agreement. However, studies have shown that the level of interobserver agreement is high for ^68^Ga-PSMA PET/CT^(33-35)^ and moderate for MRI^(36,37)^ . Our study also has some strengths. We were able to collect clinical, mpMRI, ^68^Ga-PSMA PET/CT, and histopathological data for a total of 65 patients. All patients underwent mpMRI and ^68^Ga-PSMA PET/CT before surgery, which is not usual in clinical practice given the cost of those examinations. In addition, all of the patients were evaluated by the same surgical group, which also performed all of the surgical procedures, thus ensuring homogeneity in the therapeutic decision-making process. Furthermore, all mpMRI scans were reviewed by the same radiologist, and all ^68^Ga-PSMA PET/CT scans were reviewed by the same nuclear physician, which made the assessment uniform.

In conclusion, ^68^Ga-PSMA PET/CT and mpMRI appear to perform similarly in terms of their ability to localize a tumor in the prostate. Therefore, ^68^Ga-PSMA PET/CT is a promising tool for detecting and evaluating the primary tumor, which can alter the staging and management of prostate cancer.
